# Milk‐Derived Kefir Improves Cardiac Function in Female Rats Subjected to Myocardial Infarction

**DOI:** 10.1155/jnme/9655398

**Published:** 2026-08-12

**Authors:** Ávila Iglesias Caliari, Juarez Henrique Ferreira, Licia Cristina Silva de Lima Oliveira, Tadeu Uggere de Andrade, Ewelyne Miranda de Lima, Simone Alves de Almeida, Girlandia Alexandre Brasil

**Affiliations:** ^1^ Department of Pharmaceutical Sciences, Vila Velha University–UVV, Vila Velha, Espírito Santo, Brazil; ^2^ Department of Physiology and Pharmacology, Health Sciences Center, Federal University of Espírito Santo–UFES, Vitória, Espírito Santo, Brazil

**Keywords:** cardiac function, cardiac remodeling, collagen deposition, oxidative stress, probiotic

## Abstract

Cardiovascular diseases remain a major global health burden, with myocardial infarction (MI) being a leading cause of mortality and long‐term disability. Kefir, a fermented milk beverage rich in probiotic and bioactive compounds, has demonstrated cardioprotective effects in experimental models of hypertension. However, its effects in MI induced by coronary artery ligation remain poorly understood. This study investigated the impact of chronic kefir administration on cardiac function and remodeling in female Wistar rats subjected to MI. Animals were allocated to three groups: sham‐operated (S), infarct vehicle‐treated (IV), and infarct kefir‐treated (IK). Treatments were administered daily for 60 days. Hemodynamic parameters, infarct area, collagen deposition, oxidative stress markers, and morphometric changes were evaluated. Chronic kefir treatment significantly reduced left ventricular end‐diastolic pressure (LVEDP), infarct area, and collagen deposition in infarcted animals. Kefir also improved hemodynamic parameters by reducing systolic, diastolic, and mean arterial pressures while increasing heart rate. In addition, oxidative stress was attenuated, as evidenced by lower levels of advanced oxidation protein products (AOPP). No evidence of systemic toxicity or organ hypertrophy was observed. These findings suggest that milk‐derived kefir exerts cardioprotective effects by improving hemodynamic function, attenuating adverse cardiac remodeling, and reducing oxidative stress. Collectively, the results support the potential of kefir as an adjunctive strategy for improving cardiac recovery following MI.

## 1. Introduction

Cardiovascular diseases (CVDs) are the leading cause of death worldwide, with Brazil being one of the 70 countries where CVDs represent the primary cause of mortality [1–3]. Among these conditions, acute myocardial infarction (MI) is one of the most prevalent CVDs and is responsible for a significant number of hospitalizations and deaths globally [4, 5]. Patients who experience MI often develop substantial limitations in daily activities and may subsequently progress to heart failure or renal dysfunction conditions associated with a poor prognosis and increased mortality [6].

Etiologically, MI is caused by an interruption in blood flow through the coronary arteries, leading in myocardial ischemia, adverse cardiac remodeling, and ultimately cardiomyocyte death, which impairs cardiac function and ventricular pressure regulation [7, 8]. Several risk factors contribute to the development of MI, categorized into modifiable and nonmodifiable factors. Modifiable risk factors include dyslipidemia, hypertension (often managed with medication), alcohol consumption, smoking, physical inactivity, and an unhealthy diet, all of which can be addressed through lifestyle changes [9, 10]. Conversely, nonmodifiable factors include age, sex, and genetic predisposition [9]. MI can occur in individuals with preexisting cardiovascular conditions or as their first coronary event [7, 11].

The diagnosis of MI is typically based on the electrocardiographic findings, elevated levels of myocardial necrosis biomarkers, and imaging assessments [7]. Once diagnosed, patients require intensive medical care and long‐term pharmacological therapy, often involving multiple drugs administered individually or in combination, depending on disease severity and complications [12, 13]. However, these pharmacological treatments frequently cause adverse side effects, which may lead to treatment discontinuation, resulting in further health deterioration [14].

Given the limitations and adverse effects associated with conventional drug therapies, there is increasing interest in alternative treatments that can mitigate disease progression while minimizing undesired side effects [15]. Among such alternatives, probiotics have gained attention due to their beneficial effects on gut homeostasis, modulating gut microbial composition and metabolic activity, including cardiovascular protection. Notably, probiotics have been linked to blood pressure reduction, a key risk factor associated with MI [16–19].

Kefir, a fermented milk beverage containing a complex consortium of bacteria and yeasts, has attracted increasing scientific interest because of its potential health benefits [20–22]. Kefir exhibits cardioprotective properties, including blood pressure reduction [23, 24], decreased collagen deposition [25], infarct area reduction, and lowered enzymatic markers of cardiac injury (AST, CK, and LDH) [26]. Additionally, kefir has been shown to modulate infarction‐related signaling pathways and exert anti‐inflammatory and antioxidant effects [20, 26, 27].

It is well established that MI directly increases reactive oxygen species (ROS) levels through specific enzymatic pathways and cardiac signaling mechanisms. The antioxidant potential of kefir in acute myocardial infarction (AMI) models has been demonstrated in studies using Wistar rats, where pretreatment with milk‐fermented kefir exhibited cardioprotective effects against isoproterenol‐induced MI, a well‐known *β*‐adrenergic agonist [26]. However, the effects of kefir treatment in an experimental model of MI induced by coronary artery ligation remain unexplored. Nevertheless, the mechanisms underlying the cardioprotective effects of kefir following MI remain incompletely understood.

Although previous studies have demonstrated beneficial cardiovascular effects of kefir in spontaneously hypertensive rats (SHRs), including improvements in endothelial function, autonomic regulation, and blood pressure control [23, 24], as well as protection against isoproterenol‐induced myocardial injury [26], its effects in a clinically relevant model of MI induced by permanent coronary artery ligation remain unknown. Furthermore, evidence regarding the effects of chronic kefir treatment in female animals following MI is scarce. To the best of our knowledge, this is the first study to evaluate the effects of chronic kefir treatment initiated after MI induction in female rats subjected to permanent coronary artery ligation, a model that reproduces key features of ischemic myocardial injury and postinfarction ventricular remodeling more closely than pharmacologically induced myocardial injury models [27, 28].

Accordingly, we hypothesized that chronic kefir treatment would attenuate adverse postinfarction cardiac remodeling and improve cardiac function, at least in part, through mechanisms associated with reduced oxidative stress. To test this hypothesis, we evaluated hemodynamic parameters, infarct area, collagen deposition, oxidative stress markers, and morphometric characteristics in female rats subjected to MI induced by permanent coronary artery ligation.

## 2. Materials and Methods

### 2.1. Kefir Preparation

Kefir was prepared following the method described by Santanna et al. [29], adhering to the traditional preparation process commonly used by the population. The fermentation protocol employed in the present study has been previously characterized microbiologically and chemically by Leite et al. [30], who reported the presence of lactic acid bacteria, acetic acid bacteria, and yeasts, as well as the production of fermentation‐derived organic acids. These findings are consistent with those reported by Friques et al. [31], who identified a complex microbial consortium in Brazilian kefir grains, including *Lactobacillus* spp., *Acetobacter* spp., *Enterococcus* spp., *Leuconostoc* spp., and yeasts, produced using the same traditional fermentation method. Although the microbiological and biochemical composition of the specific batches used in the present study was not directly assessed, all preparations were produced under standardized conditions following previously validated protocols. Briefly, kefir grains were incubated with pasteurized cow’s milk (*Fiori*, Santa Tereza, ES) at a 4% (w/v) ratio for 24 h at room temperature. After incubation, the grains were separated using a plastic sieve, and the resulting fermented milk was refrigerated at approximately 8°C for 24 h. Fresh kefir was prepared daily for animal treatment.

### 2.2. Experimental Animals

Female Wistar rats (*Rattus norvegicus*), two months old and weighing between 150 and 200 g, were used in the study. The animals were obtained from the Experimental Monitoring Laboratory of Vila Velha University and housed in groups of five per cage under controlled temperature and humidity conditions, with a 12/12‐h light/dark cycle. Water and food were provided *ad libitum*. All experimental procedures were approved by the Animal Ethics Committee of Vila Velha University (CEUA‐UVV: 464/2018).

### 2.3. MI Induction

Animals were anesthetized with ketamine and xylazine (70/7 mg/kg, intraperitoneally) for infarction induction. Animals were placed in the supine position, and the left thoracic region was shaved and cleaned with 70% ethanol. A skin incision was made to expose the intercostal muscles, and a subsequent incision at the fourth intercostal space exposed the heart. The left anterior descending coronary artery was ligated using a previously placed purse‐string suture with nontraumatic mononylon thread (6.0 *Ethicon*). After ligation, the heart was repositioned, and the thoracic cavity was immediately closed. The animals were monitored using an electrocardiogram device (FE221 Bridge Amp, ADInstruments, Australia) and subjected to mechanical ventilation if necessary. The sham‐operated group (SHAM) underwent the same surgical procedures without coronary artery ligation [32].

### 2.4. Experimental Protocol

Animals were randomly assigned to the following groups: Sham group (S): Underwent sham surgery and received only the kefir vehicle (pasteurized Type C milk; 0.3 mL/100 g body weight); Infarction vehicle group (IV): Underwent infarction surgery and received the kefir vehicle (pasteurized Type C milk; 0.3 mL/100 g body weight); Infarction kefir group (IK): Underwent infarction surgery and received kefir daily at 0.3 mL/100 g body weight [23].

All treatments were initiated 24 h postsurgery and continued for 60 days. Subsequently, animals underwent hemodynamic evaluations.

### 2.5. Hemodynamic Assessments

Twenty‐four hours after the last treatment, animals were anesthetized with ketamine and xylazine (100/10 mg/kg, intraperitoneally) for hemodynamic evaluations. The right cervical region was shaved and disinfected with 70% ethanol. An incision was made to expose the right carotid artery. A polyethylene catheter (PE‐50) filled with heparinized saline (0.9% NaCl; 50 IU/mL) was inserted and secured with cotton thread. The catheter was connected to a pressure transducer (FE221 Bridge Amp, ADInstruments, Australia) linked to a data acquisition system (PowerLab 4/35, ADInstruments, Australia).

After stabilization, the following parameters were recorded: heart rate (HR), systolic blood pressure (SBP), diastolic blood pressure (DBP), and mean arterial pressure (MAP). The catheter was then advanced into the left ventricle to measure cardiac function parameters: left ventricular end‐diastolic pressure (LVEDP), maximum and minimum pressure derivatives (+dP/dt_max_ and −dP/dt_min_), and Tau. Postassessment, the catheter was retracted to the carotid artery to verify potential aortic valve damage, indicated by a pressure drop of more than 5 mmHg. Animals presenting a pressure decrease greater than 5 mmHg were excluded from the analysis. No animals exhibited evidence of aortic valve damage.

### 2.6. Histological Analysis

Following hemodynamic assessments, animals were euthanized by exsanguination, and organs were collected. The hearts were cleaned, dried, and weighed. They were sectioned into three parts and preserved in buffered formalin for histological analysis. Sections were processed and cut into 5 µm sections, mounted on glass slides, and stained with hematoxylin and eosin (H&E) for morphometric analysis or Picrosirius Red for infarct areas and collagen deposition assessment.

### 2.7. Infarct Area Assessment

Infarct area analysis was performed through morphometric evaluation. Digital images of the stained sections were acquired using a QImaging Retiga 2000R camera and analyzed using the open‐source software ImageJ. Infarcted regions were identified using Picrosirius Red staining and quantified relative to the total myocardial area [32].

### 2.8. Morphometric and Collagen Deposition Assessment

To quantify tissue collagen, slides were analyzed under an optical microscope, and ten images from each slide were collected. The percentage of red staining was quantified using ImageJ. Morphometric analysis of cardiomyocyte hypertrophy was performed indirectly by measuring nuclear area, length, and width [33].

### 2.9. Ponderal Data

For organ weight analysis, the right tibia was carefully dissected, ensuring the proximal and distal portions remained intact. The bone was cleaned of muscle tissue and measured. Organs (kidney, liver, heart, and adrenal glands) were collected, cleaned, dried, and weighed. Hypertrophy was assessed based on the organ‐to‐tibia length ratio [34, 35].

### 2.10. Oxidative Stress Markers

Protein and lipid oxidation were assessed using advanced oxidation protein products (AOPP) and thiobarbituric acid reactive substances (TBARS) assays. For AOPP evaluation, the method of Witko‐Sarsat et al. [36] was used. The heart homogenate was mixed with potassium iodide (KI, 1.16 M) and acetic acid. After continuous agitation, the absorbance was measured at 340 nm (Filter Max F5 Multi‐Mode Microplate Readers, Molecular Devices, California, EUA). The result was determined using a standard curve with chloramine *T* (100–1000 μM) and was expressed as μM de chloramine T/mg protein. To determine TBARS, the cardiac homogenate (200 μL) was mixed with thiobarbituric acid (200 μL). After heating (100°C) for 2 h and cooling to room temperature, the absorbance was measured at 532 nm (Filter Max F5 Multi‐Mode Microplate Readers). A standard curve with malondialdehyde was obtained (MDA; 0.5–25 μM), and the results were expressed as μM of MDA/mg protein.

### 2.11. Statistical Analysis

Data are presented as mean ± standard error of mean (SEM). Normality was assessed using the Kolmogorov–Smirnov test, and homogeneity of variances was evaluated using the Brown–Forsythe test. All datasets satisfied the assumptions required for parametric analyses. Since all outcome variables were assessed at a single experimental endpoint after 60 days of treatment, repeated‐measures analyses were not applicable. The infarct area was analyzed using an unpaired Student’s *t*‐test, whereas all other variables were analyzed using one‐way analysis of variance (ANOVA) followed by Tukey’s post hoc test. To complement statistical significance testing, effect sizes were calculated as eta squared (*η* ^2^) for ANOVA models and Cohen’s *d* for pairwise comparisons. Statistical significance was set at *p* < 0.05. GraphPad Prism 10 was used for analysis.

## 3. Results

This study investigated the effects of chronic milk‐derived kefir treatment in a rat model of MI induced by coronary artery ligation. The analyses focused on hemodynamic parameters, infarct area, collagen deposition, oxidative stress markers, and morphometric parameters.

### 3.1. Hemodynamic Parameters

**FIGURE 1:**
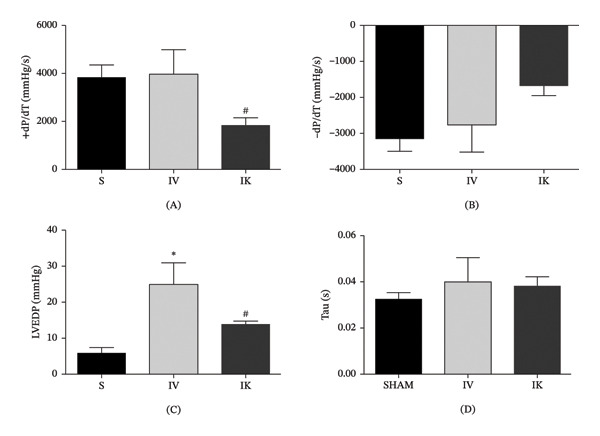
Hemodynamic assessment of infarcted rats chronically treated with kefir. (A, B) Maximum and minimum pressure derivatives (±dP/dt), respectively. (C) Left ventricular end‐diastolic pressure (LVEDP). (D) Tau (time constant of isovolumic relaxation). S: sham group; IV: infarcted vehicle group; IK: infarcted kefir‐treated group. Data are presented as mean ± SEM (*n* = 8 per group). Statistical analysis was performed using one‐way ANOVA, followed by Tukey’s post hoc test. ^∗^ *p* < 0.05.

Chronic kefir treatment for 60 days improved cardiac function in infarcted animals, as illustrated in (Figure 1) (panels A‐D). LVEDP was significantly elevated in infarcted animals (IV: 25.02 ± 5.93 mmHg; *p* < 0.05 vs. S). However, kefir treatment normalized LVEDP values (S: 5.83 ± 1.62 mmHg; IK: 13.84 ± 0.85 mmHg; *p* > 0.05 vs. S).

No significant differences were observed for Tau (S: 0.032 ± 0.002; IV: 0.040 ± 0.010; IK: 0.038 ± 0.03 s) and −dP/dt_min_ (S: −3139 ± 373; IV: −2768 ± 757; IK: −1677 ± 276 mmHg/s). However, a significant reduction in +dP/dt_max_ was observed in the kefir‐treated group (S: 3825 ± 522; IV: 3956 ± 1022; IK: 1831 ± 332 mmHg/s).

Kefir treatment was associated with lower +dP/dtmax values and normalization of LVEDP compared with the infarct vehicle group. Notably, infarcted animals treated with the vehicle did not exhibit improvements, confirming the persistence of ventricular dysfunction in this group.

Additional hemodynamic parameters, including SBP, DBP, and MAP, were significantly reduced in kefir‐treated animals, while HR was increased in this group (Table 1).

**TABLE 1:** Results of hemodynamics evaluation in animals submitted to myocardial infarction and 8 weeks of kefir treatment.

	Groups		
	S	IV	IK
SBP	149.5 ± 9.5	125.8 ± 8.8	112.8 ± 7.2^∗^
DBP	108.0 ± 6.2	95.1 ± 6.1	80.6 ± 12.0^∗^
MAP	125.9 ± 6.6	107.9 ± 6.2	96.7 ± 9.2^∗^
HR	226.8 ± 17.0	226.4 ± 21.8	303.6 ± 26.6^∗#^

*Note:* Data are present as mean ± standard error of mean. All data were evaluated by one‐way ANOVA, followed by Tukey’s post hoc test. Results were considered significantly different when *p* < 0.05.

^∗^ *p* < 0.05 compared to the S group.

^#^ *p* < 0.05 compared to the IV group.

### 3.2. Infarct Area and Collagen Deposition

**FIGURE 2:**
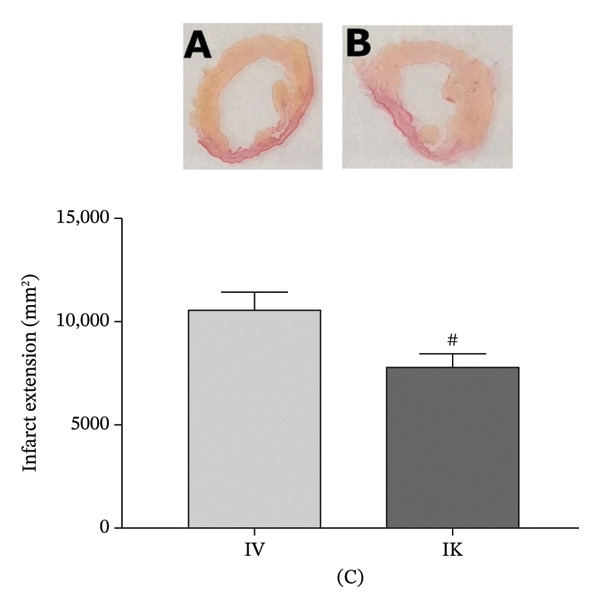
Assessment of infarct size in kefir‐treated animals. (A, B) Representative images of the cardiac infarct area from vehicle‐treated (A) and kefir‐treated (B) animals. (C) Quantification of infarct extension in both groups. IV: Infarcted vehicle group; IK: infarcted kefir‐treated group. Data are presented as mean ± standard error of the mean (SEM) (*n* = 8 per group). Statistical analysis was performed using an unpaired *t*‐test. ^#^ *p* < 0.05.

**FIGURE 3:**
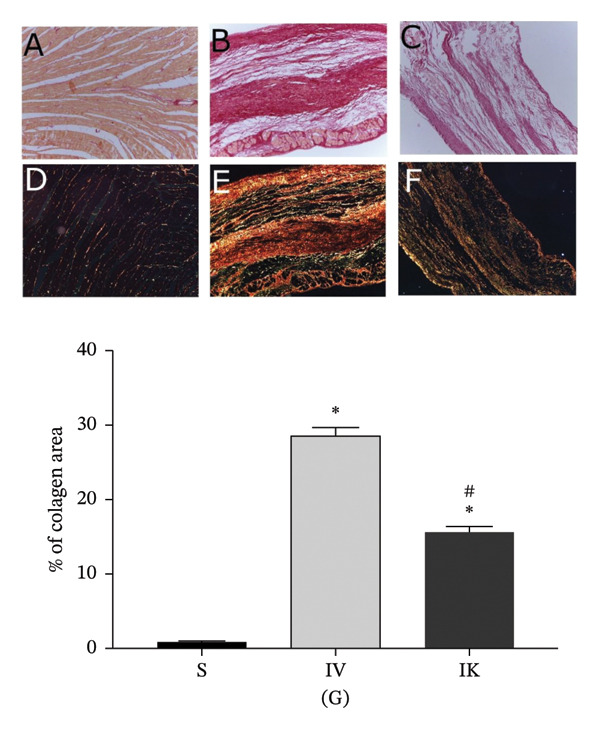
Assessment of collagen deposition in kefir‐treated animals. Representative images of cardiac tissue under brightfield (A–C) and polarized light (D–F), respectively. (G) Quantification of collagen content in animals from different groups. S: sham group; IV: infarcted vehicle group; IK: infarcted kefir‐treated group. Data are presented as mean ± standard error of the mean (SEM) (*n* = 8 per group). Statistical analysis was performed using one‐way ANOVA, followed by Tukey’s post hoc test. ^∗^ *p* < 0.05; ^#^ *p* < 0.05.

Histological analysis using Picrosirius Red staining (Figures 2 and 3) revealed that kefir treatment significantly reduced infarct areas by 26.4% compared to the infarct vehicle group (IV: 10,531 ± 922.6 mm^2^; IK: 7749 ± 695.5 mm^2^).

This reduction in the infarct area was accompanied by a 47.79% decrease in collagen deposition in the hearts of infarcted animals (S: 1.079 ± 0.09%; IV: 28.56 ± 1.23%; IK: 14.91% ± 1.59%). These findings indicate that kefir can attenuate pathological remodeling processes following AMI.

Effect size analyses confirmed the biological relevance of the observed treatment effects. The largest effects were observed for collagen deposition, infarct area, and LVEDP, whereas large effects were also detected for several hemodynamic parameters. Detailed effect size estimates are presented in Supporting Table S1.

### 3.3. Oxidative Stress Markers

**FIGURE 4:**
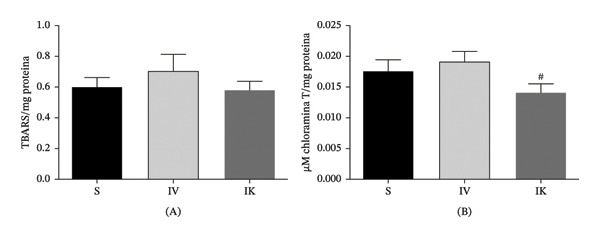
Effect of kefir on oxidative stress markers in myocardial tissue after infarction. (A) TBARS: thiobarbituric acid reactive substances. (B) AOPP: advanced oxidation protein products. S: sham group; IV: infarcted vehicle group; IK: infarcted kefir‐treated group. Data are presented as mean ± SEM (*n* = 8 per group). Statistical analysis was performed using one‐way ANOVA, followed by Tukey’s test. ^#^ *p* < 0.05.

Oxidative stress markers, including AOPP and TBARS, were analyzed (Figure 4). No significant changes were observed in TBARS levels among the groups. However, AOPP levels were significantly lower in the kefir‐treated group than in the infarct vehicle group (Panel B).

### 3.4. Morphometric Parameters

No significant differences were observed in morphometric parameters related to cardiac hypertrophy (Supporting Table S2). Similarly, no significant differences were observed in organ weight‐to‐tibia length ratios among the experimental groups (kidney, lung, heart, and adrenal glands) (Supporting Table S3).

## 4. Discussion

This study demonstrated, for the first time, that chronic treatment with milk‐derived kefir improves cardiac function and attenuates adverse postinfarction remodeling in female rats subjected to permanent coronary artery ligation. Previous studies evaluating kefir have focused primarily on hypertension models or pharmacologically induced myocardial injury [23, 24, 26, 37], whereas the present study investigated a clinically relevant model of MI characterized by ischemic tissue loss and chronic ventricular remodeling.

Our data showed an increase in basal HR, which is an unusual finding for interventions that promote cardioprotection, and this should be taken into account when considering the use of kefir after MI. However, this increase did not appear to compromise the beneficial effects observed following kefir administration. Additionally, a significant reduction in systolic, diastolic, and MAPs observed in the kefir‐treated group (IK) suggests a direct influence of kefir on cardiovascular function.

This effect is likely associated with bioactive compounds produced during kefir fermentation, including peptides with angiotensin‐converting enzyme (ACE) inhibitory properties [20, 21, 38–40]. These bioactive peptides can reduce the conversion of Angiotensin I to Angiotensin II, a key process in blood pressure regulation [41]. Such a mechanism mirrors the action of pharmacological ACE inhibitors, supporting the observed reduction in blood pressure parameters in the kefir‐treated group compared to the infarct vehicle group.

Previous studies have also shown the beneficial effects of kefir on blood pressure reduction and endothelial function improvement in SHRs [22, 23]. The regulation of gut integrity by kefir may contribute to these effects by reducing neuroinflammation in brain regions that control cardiovascular function, such as the paraventricular nucleus (PVN) and the rostral ventrolateral medulla (RVLM) [24]. This anti‐inflammatory action, characterized by the suppression of proinflammatory cytokines in these regions, could explain the reduced arterial pressures observed in this study.

Interestingly, kefir‐treated animals exhibited a significant increase in HR despite the concomitant reduction in arterial pressure. Although the mechanisms responsible for this response were not investigated in the present study, it may reflect compensatory cardiovascular adjustments aimed at maintaining cardiac output in the setting of reduced peripheral vascular resistance. Previous studies have shown that kefir improves baroreflex sensitivity and modulates cardiac autonomic control in hypertensive rats [16] and may reduce blood pressure through gut–brain pathways involved in autonomic regulation [24]. Therefore, the increased HR observed herein may represent an adaptive autonomic response secondary to blood pressure reduction. Importantly, this finding was not accompanied by worsening ventricular function or adverse cardiac remodeling. In contrast, kefir‐treated animals displayed lower LVEDP, reduced infarct size, decreased collagen deposition, and lower AOPP levels, suggesting an overall cardioprotective profile. Nevertheless, further studies assessing autonomic balance, baroreflex activity, and myocardial oxygen demand are necessary to clarify the physiological significance of this finding.

The reduction of the infarct area and collagen deposition in the kefir‐treated group indicates a cardioprotective effect of kefir. Excessive collagen deposition is a hallmark of pathological cardiac remodeling following MI and contributes to reduced myocardial elasticity and impaired ventricular function [9, 42]. The observed reduction in collagen deposition in the kefir‐treated group may be related, at least in part, to modulation of matrix metalloproteinase (MMP) activity, enzymes responsible for degrading the extracellular matrix, including collagen [43, 44].

The potential influence of kefir on the renin–angiotensin–aldosterone system (RAAS) also plays a role in these outcomes. Angiotensin II (Ang II) is known to promote oxidative stress and inflammatory responses that exacerbate cardiac remodeling [45]. By inhibiting ACE, kefir likely reduces Ang II levels, mitigating these pathological processes. Additionally, kefir’s antioxidant properties contribute to reducing ROS, preventing further tissue damage [46, 47].

These findings are supported by the observation that kefir treatment significantly reduced oxidative stress markers, particularly AOPP, indicating a protective effect against protein oxidation. Although no significant effects were observed for lipid peroxidation markers (TBARS), the reduction in protein oxidative stress aligns with previous studies demonstrating kefir’s antioxidant capacity [29]. Kefir’s ability to enhance endogenous antioxidant defenses, including catalase, superoxide dismutase (SOD), and glutathione (GSH), may explain this protective effect. These enzymes play crucial roles in neutralizing ROS, preventing oxidative damage to cardiac tissues [48, 49].

The protective role of kefir against oxidative stress was further supported by studies showing its ability to reduce ROS levels and improve cellular antioxidant status [50, 51]. These findings suggest that kefir may contribute to reducing oxidative damage post‐MI, aiding in cardiac recovery.

Although the present study did not directly characterize the microbiological composition or metabolite profile of the kefir preparation, previous studies using similar fermentation protocols have identified complex microbial communities and fermentation‐derived metabolites in Brazilian kefir preparations [16, 30, 31]. Previous studies have also shown that kefir can modulate oxidative stress [26], improve endothelial function [16], and influence cardiovascular regulatory pathways, including the renin–angiotensin system [23, 24]. Therefore, the mechanistic interpretations proposed herein should be considered hypothesis‐generating and require confirmation in future studies specifically designed to characterize kefir‐derived bioactive compounds.

Taken together, the findings of the present study suggest that the cardioprotective effects of kefir are primarily associated with attenuation of oxidative stress and pathological cardiac remodeling. The reduction in AOPP levels, infarct area, collagen deposition, and LVEDP supports a mechanism involving preservation of myocardial structure and function following ischemic injury. Although previous studies have suggested additional actions of kefir on endothelial function, autonomic regulation, and gut microbiota‐related pathways [16, 23, 24], these mechanisms were not directly evaluated in the present study and therefore should be interpreted cautiously.

Our morphometric analysis revealed no significant changes in organ weight‐to‐tibia ratios. Although a numerical increase in adrenal gland weight was observed in the kefir‐treated group, this difference did not reach statistical significance. Importantly, Ogawa et al. [52] also reported no significant alterations in organ weight or tissue morphology following treatment with *Lactobacillus kefiranofaciens* M1, a strain derived from kefir. Overall, the absence of hypertrophic alterations and consistent organ weights confirm the safety of chronic kefir treatment.

The observed reduction in infarct area and collagen deposition, together with improved hemodynamic parameters and lower AOPP levels, suggests that kefir attenuates postinfarction cardiac remodeling. Although inflammatory mediators were not evaluated in the study, previous investigations have reported anti‐inflammatory effects of kefir, including reductions in cytokine production and atherosclerotic lesion progression [52–54]. Therefore, while inflammatory modulation may contribute to the cardioprotective effects observed herein, this mechanism was not directly assessed and should be interpreted cautiously.

Consistent with these findings, Mert et al. [26] demonstrated that kefir administration reduced biochemical markers of cardiac injury and oxidative stress in a model of isoproterenol‐induced MI. The authors also reported increased GSH levels and reduced malondialdehyde and AOPP concentrations, further supporting the antioxidant and cardioprotective properties of kefir.

### 4.1. Limitation of the Study

A limitation of the present study is that only female rats were evaluated, precluding direct comparisons between sexes. Therefore, it is not possible to determine whether the cardioprotective effects of kefir observed herein are sex‐specific. Future studies directly comparing male and female animals under identical experimental conditions are warranted to clarify potential sex‐specific differences in the response to kefir treatment following MI.

Another limitation is the lack of microbiological and biochemical characterization of the kefir preparation used throughout the experimental protocol. Although kefir was produced using a standardized and previously validated fermentation procedure, variations in microbial composition and metabolite production may occur between preparations. Therefore, future studies should incorporate microbial profiling and metabolomic analyses to identify the bioactive compounds and mechanistic pathways underlying the cardioprotective effects observed herein.

## 5. Conclusion

Overall, the findings of this study demonstrate that chronic treatment with milk‐derived kefir improves cardiac function and attenuates adverse postinfarction remodeling in female rats subjected to MI. These effects were associated with reductions in the infarct area, collagen deposition, LVEDP, and protein oxidative stress. Collectively, these findings suggest that kefir may contribute to preserving myocardial structure and function following ischemic injury. However, further studies are required to elucidate the precise molecular mechanisms and bioactive compounds underlying these cardioprotective effects. Although translation to clinical settings requires additional investigation, the present findings support the potential of kefir as an adjunctive strategy for improving recovery following MI.

## Funding

This work was supported by the Fundação de Amparo à Pesquisa do Espírito Santo (EDITAL CNPq/FAPES N° 22/2018, grant 034/2019). This study was financed in part by Coordenação de Aperfeiçoamento de Pessoal de Nível Superior‐Brasil (CAPES)‐Finance Code 001.

## Conflicts of Interest

The authors declare no conflicts of interest.

## Supporting Information

Additional supporting information can be found online in the Supporting Information section.

## Supporting information


**Supporting Information** Supporting Information is provided to support the findings of this study. SupportingTable S1 presents effect size analyses (*η* ^2^ and Cohen’s d) for the main outcomes. Supporting Table S2 summarizes morphometric parameters related to cardiac hypertrophy, whereas Supporting Table S3 presents organ weight‐to‐tibia length ratios.

## Data Availability

The data that support the findings of this study are available from the corresponding author upon reasonable request.
