# Supplementary material for: Milk‐Derived Kefir Improves Cardiac Function in Female Rats Subjected to Myocardial Infarction
Source: J Nutr Metab. 2026 Aug 12;2026:9655398. doi: 10.1155/jnme/9655398 (PMC13469750; doi:10.1155/jnme/9655398)
Supplement: Supplementary file 1 [file JNME-2026-9655398-s001.docx]

**Supplementary Table S1. Effect size estimates for the main outcomes evaluated in infarcted animals treated with kefir.**

| **Variable** | **η²** | **Cohen's d (IV vs IK)** | **Magnitude** |
| --- | --- | --- | --- |
| LVEDP | 0.558 | -1.18 | Very large |
| SBP | 0.421 | -0.72 | Large |
| MAP | 0.389 | -0.64 | Medium-to-large |
| HR | 0.387 | 1.42 | Very large |
| +dP/dt_max_ | 0.317 | -1.25 | Very large |
| PAD | 0.300 | -0.68 | Medium-to-large |
| -dP/dt_min_ | 0.263 | 0.86 | Large |
| Tau | 0.059 | -0.11 | Negligible |
| Infarct area | — | -1.70 | Very large |
| Collagen deposition | 0.960 | -4.81 | Extremely large |

**Interpretation of effect sizes:**

For η², values of 0.01, 0.06, and 0.14 indicate small, medium, and large effects, respectively (Cohen, 1988). For Cohen's d, values of 0.2, 0.5, 0.8, and >1.2 indicate small, medium, large, and very large effects, respectively. Values above 2.0 were classified as extremely large effects.

| **Supplementary Table S2.** Data of morphometric analysis from cardiomyocytes | | | |
| --- | --- | --- | --- |
|  | Groups | | |
|  | S | IV | IK |
| Number | 4.03±0.62 | 2.73±0.54 | 2.87±0.17 |
| Area (mm^2^) | 11773±633.7 | 11390±427.3 | 11818±942.3 |
| Length (mm^2^) | 233.4±13.5 | 229.0±6.55 | 228.9±12.5 |
| Width (mm^2^) | 53.89±2.16 | 60.1±0.87 | 59.4±1.8 |
| Data are present as mean ± standard error of mean. All data was evaluated by one-way ANOVA, followed by Tukey’s post hoc test. Result were considered different when p<0.05. | | | |

| **Supplementary Table S3.** Ponderal data from animals submitted to myocardial infarction and kefir treatment for eight weeks | | | |
| --- | --- | --- | --- |
|  | **Groups** | | |
|  | S | IV | IK |
| Kidney/tibial length (mg/mm) | 0.022±0.001 | 0.021±0.001 | 0.022±0.001 |
| Lung /tibial length (mg/mm) | 0.259±0.134 | 0.256±0.018 | 0.283±0.012 |
| Heart /tibial length (mg/mm) | 0.0178±0.001 | 0.0182±0.001 | 0.0198±0.001 |
| Adrenal /tibial length (mg/mm) | 0.0006±0.0001 | 0.0006±0.0002 | 0.0011±0.0003 |
| Data are present as mean ± standard error of mean, the results were submitted to one-way ANOVA followed by Tukey’s post hoc test. The result was considered different when p<0.05. | | | |
